# Supplementary material for: Work-related musculoskeletal disorder and its associated factors among bank workers in Ethiopia: A systematic review and meta-analysis
Source: PLoS One. 2025 May 16;20(5):e0323958. doi: 10.1371/journal.pone.0323958 (PMC12083818; doi:10.1371/journal.pone.0323958)
Supplement: S2 Table — (DOCX) [file pone.0323958.s002.docx]

**Results of JBI Quality Assessment**

| Included articles | Criteria | | | | | | | | | |
| --- | --- | --- | --- | --- | --- | --- | --- | --- | --- | --- |
|  | Clear eligibility criteria | Description of study subject and study setting | Valid & reliable method to measure the exposure | Standard criteria used for measurement of the condition | Identification of confounding factors | Development of strategies to deal with confounding factors | Valid and reliable method to measure outcomes | Appropriate statistical analysis | Total score out of 8 | Quality Score (%) |
| Dagne *et al* | Yes | Yes | Yes | Yes | Yes | Yes | Yes | Yes | 8 | 100 |
| Temesgen *et al* | No | Yes | No | Yes | Yes | Yes | Yes | Yes | 6 | 75 |
| Workneh and Mekonen | No | Yes | Yes | Yes | Yes | Yes | Yes | Yes | 7 | 87.5 |
| Jonga *et al* | Yes | Yes | No | Yes | Yes | Yes | Yes | Yes | 7 | 87.5 |
| Etana *et al* | Yes | Yes | Yes | Yes | Yes | Yes | Yes | Yes | 8 | 100 |
| Kibret *et al* | Yes | Yes | Yes | Yes | Yes | Yes | Yes | Yes | 8 | 100 |
| Demissie et al | Yes | Yes | Yes | Yes | Yes | Yes | Yes | Yes | 8 | 100 |
| Demissie et al | Yes | Yes | No | Yes | Yes | Yes | Yes | Yes | 7 | 87.5 |
