# Supplementary material for: Work-related musculoskeletal disorder and its associated factors among bank workers in Ethiopia: A systematic review and meta-analysis
Source: PLoS One. 2025 May 16;20(5):e0323958. doi: 10.1371/journal.pone.0323958 (PMC12083818; doi:10.1371/journal.pone.0323958)
Supplement: Attachment — Submitted filename: Point by Point Response to Editors and Reviewers.docx [file pone.0323958.s006.docx]

**Date: 4 March 2025**

**To Esteemed Editor/s and Reviewers**

**Subject: Response to Editors’ and Reviewers’ Comments and Suggestions**

It is our great pleasure to thank the editors and reviewers of our manuscript entitled **“Work-related Musculoskeletal disorder and its associated factors among bank workers in Ethiopia: A systematic review and meta-analysis”** for the valuable comments and/or suggestions. Upon conducting a comprehensive review of the comments, suggestions and questions provided and asked, we have meticulously addressed each one. We are confident that the revisions we’ve made meet your expectations and significantly improve the quality of our manuscript. To aid in the review process, we have prepared two versions of the manuscript for your convenience: one with clear, easy to read copy and the other highlighted with track-changes showing the changes made from previously submitted version of the manuscript. These versions aim to facilitate understanding and transparency regarding the modifications made. We are pleased to present the revised manuscript along with our responses to the comments. We eagerly await your favorable consideration for publication.

**With Regards!**

**Authors**

**Part-I: Authors Response to Editor(s)’ Comments/Suggestions**

| **Editor(s)’ Comments/Suggestions to Authors** | | **Authors’ Response** |
| --- | --- | --- |
| **#1** | After careful consideration, we feel that it has merit but does not fully meet PLOS ONE’s publication criteria as it currently stands. Therefore, we invite you to submit a revised version of the manuscript that addresses the points raised during the review process. | Thank you for the invitation to respond to the reviewers' comments and revise the manuscript. We have thoroughly addressed the suggestions and are pleased to submit the revised version for your consideration. |
| **#2** | While both reviewers find merit in your manuscript, they have raised several important feedback and comments. I also believe that those comments are very useful to improve the quality of the manuscript. You should revise your manuscript based on reviewer and editor comments. | Thank you for your insightful guidance. We revised our manuscript as per the comments given by the editor and reviewers. |
| **#3** | There are many grammatical errors throughout the manuscript. I would suggest for a language editing by a native speaker. | Thank you very much for your suggestion. |
| **#4** | Authors should add statistics to support their claim in the abstract. | Thank you once more. We corrected it. |
| **#5** | Please confirm if risk of bias was assessed? how? | Thank for the question. We assessed the risk of bias using the Joanna Briggs Institute (JBI) tools based on the study design of each article.  For example, articles having a JBI quality score of greater than 75% is considered to have low risk of bias.  Moreover, publication bias was assessed by funnel plot and Eggers’ test statistics. |
| **#6** | Authors stated that the article was searched by 4 reviewers, screened by 3 reviewers, and the quality was assessed by 2 reviewers. Why were all reviewers not utilized in all the stages? Whether inter-reviewer reliability was tested in any stages? what was the results? | We are very grateful for your question. At first, four reviewers were involved in searching to address many databases. Utilizing the listed number of reviewers was purely for cascading tasks. |
| **#7** | In the result section, authors should add comparators for all listed variables in Table 2. | Thank you very much for your insightful comment. We thoroughly reviewed and corrected it as per the comment. |
| **#8** | Please include the following items when submitting your revised manuscript:   - A rebuttal letter that responds to each point raised by the academic editor and reviewer(s). You should upload this letter as a separate file labeled 'Response to Reviewers - A marked-up copy of your manuscript that highlights changes made to the original version. You should upload this as a separate file labeled 'Revised Manuscript with Track Changes'. - An unmarked version of your revised paper without tracked changes. You should upload this as a separate file labeled 'Manuscript'. | Thank you for the guiding suggestion. A rebuttal letter that responds to each point raised by the academic editor and reviewer(s) is prepared.  We also highlighted the manuscript with track changes to show where the changes made in the manuscript. Moreover, we have prepared another clean version (uploaded as the manuscript file). |

**Part-II: Authors Response to Journal’s Additional Requirements**

| **Journal’s Additional Requirements** | | **Authors’ Response** |
| --- | --- | --- |
| **#1** | 1. Please ensure that your manuscript meets PLOS ONE's style requirements, including those for file naming. The PLOS ONE style templates can be found at  <https://journals.plos.org/plosone/s/file?id=wjVg/PLOSOne_formatting_sample_main_body.pdf> and  <https://journals.plos.org/plosone/s/file?id=ba62/PLOSOne_formatting_sample_title_authors_affiliations.pdf> | Thank you very much for your guidance. We followed d it. |
| **#2** | 2. As required by our policy on Data Availability, please ensure your manuscript or supplementary information includes the following:  A numbered table of all studies identified in the literature search, including those that were excluded from the analyses.  For every excluded study, the table should list the reason(s) for exclusion.  If any of the included studies are unpublished, include a link (URL) to the primary source or detailed information about how the content can be accessed.  A table of all data extracted from the primary research sources for the systematic review and/or meta-analysis. The table must include the following information for each study:  Name of data extractors and date of data extraction  Confirmation that the study was eligible to be included in the review.  All data extracted from each study for the reported systematic review and/or meta-analysis that would be needed to replicate your analyses. | Thank you very much once more for these insightful guidance and requirements. We addressed all of these issues critically. These requirements are included in our data extraction sheet and a numbered table that shows all articles included and excluded in the review. |
| **#3** | If applicable for your analysis, a table showing the completed risk of bias and quality/certainty assessments for each study or outcome.  Please ensure this is provided for each domain or parameter assessed. For example, if you used the Cochrane risk-of-bias tool for randomized trials, provide answers to each of the signaling questions for each study. If you used GRADE to assess certainty of evidence, provide judgements about each of the quality of evidence factor. This should be provided for each outcome. | Thank you. We used JBI quality assessment tool and we attached the result as a supplementary material (Supplementary Material 2). |
| **#4** | An explanation of how missing data were handled. | We thank you again. We address this issue in our manuscript under “Data extraction procedure, study quality, and risk of bias assessment” section. |

**Part-III: Authors Response to Reviewers’ Comments/Suggestions**

|  | **Contents** | **Comments/Suggestions and Questions** | **Authors’ Response** |
| --- | --- | --- | --- |
| **#1** | **Abstract** | The study reveals in terms of the gender, job stress, physical activity, and work experience were found to be factors significantly associated with work-related musculoskeletal disorder why and what measure the author taken to end up such conclusion should be stated more in the abstract. Research on other variables is limited- please elaborate other variables related to musculoskeletal disorder. | We are very thankful for your comments. The variables included in the extraction were more than nine. However, the number of variables frequently listed in at least two of the included studies was nine. Among these listed variables, gender, job stress, physical activity, and work experience were found to be factors significantly associated with work-related musculoskeletal disorder among bank workers n Ethiopia. |
|  |  | The keywords should be formatted according to MeSH (Medical Subject Headings) terms for improved indexing. | Thank you very much for your comment. We made it. |
| **#3** | **Methods** | Systematic review and meta-analysis - The author mentioned in statistical analysis table-1, all the study are cross sectional, do the study find any longitudinal study based on Ethiopia and or greater Africa if not then stated in the methods. | We thank you very much for your insightful comment. Longitudinal studies were also our desire to include in the review and meta-analysis; but we didn’t get such articles following the literature search in Ethiopia. The scope of this study is in Ethiopia only. |
|  |  | The result table-2 BMI classification only two class overweight and underweight what about normal BMI range data- Do the normal BMI has any relation with work-related musculoskeletal disorder. | Thank you for your comment. Normal BMI is the reference category for that specific variable and we corrected and put in the table. |
|  |  | The methodology adheres to the PRISMA guidelines but would benefit from a more detailed explanation of how heterogeneity across studies was addressed. | Thank you very much for your insightful comments. The heterogeneity across the included studies was addressed by priorly using random effects meta-analysis over fixed effects meta-analysis. Moreover, it was addressed by undertaking subgroup analysis and performing sensitivity test.  We have corrected the manuscript as per this valuable comment and this explanation is included in the manuscript too. |
|  |  | While statistical tools, such as I², were used, discussing additional steps taken to minimize variability, such as subgroup analyses or sensitivity tests, would strengthen the robustness of the study. | We are very thankful for your comment. We made it in the manuscript. |
|  |  | A clearer justification for the use of the random-effects model and a more detailed description of the criteria for including and excluding studies, particularly gray literature, are also recommended. | Thank you very much for your comments once more. After a thoroughly revising our manuscript, we corrected it as per the comments. |
| **#4** | **Results** | Could the author explain more how they categorized low risk bias, unclear risk of bias. | Thank you once more. Using the Joana Brigg Institute (JBI) checklist of critical appraisal, the quality of each article was critically evaluated and articles having a quality score of ≥ 75% and between 50 to 75% are considered to have low and moderate risk of bias respectively. |
|  |  | Please avoid repetition | Thank you. We corrected it after carefully reviewing our manuscript. |
|  |  | Please check reference style throughout MS | Thank you again. We checked and corrected it following a thorough review of our manuscript. |
|  |  | Recheck Legends description is as per figure number and discussion. | Thank you very much for your comment. We rechecked it. |
| **#5** | **Discussions** | The discussion contextualizes the findings well within global and regional studies but could better highlight how these findings can inform occupational health interventions and policies in Ethiopia. Discussing practical recommendations, such as workplace ergonomic improvements or stress management programs, will enhance the applicability of the manuscript. | Thank you very much for your insightful comments. We made corrections as per the comments. |
| **#6** | **Conclusions** | Additionally, the conclusion should focus more on the implications of the findings for occupational health policies and the need for further research to include diverse regions and adopt a longitudinal design. | Thank you very much again. We made corrections as per the comments. |
| **#7** | **Summary and General Comments** | Figures and tables are informative but lack clear legends and captions, making them less accessible to the readers. Ensure that all visual elements are self-contained with sufficient explanatory details. Finally, a proofreading pass is required to address minor grammatical inconsistencies and ensure clarity throughout the text. | We are very thankful for your comments and suggestions. |
